# A Rare Tumor in a Septuagenarian Female 16 Years After Radical Mastectomy for Breast Carcinoma

**DOI:** 10.1002/ccr3.70574

**Published:** 2025-07-01

**Authors:** Mohammadhossein Rahimirad, Sara Daneshvar, Shaghayegh Rahimirad, Monireh Halimi, Akbar Sharifi

**Affiliations:** ^1^ Tuberculosis & Lung Diseases Research Center Tabriz University of Medical Sciences Tabriz Iran; ^2^ General Physician, Tuberculosis & Lung Diseases Research Center Tabriz University of Medical Sciences Tabriz Iran

**Keywords:** breast carcinoma, metachronous tumor, sarcomatoid carcinoma, spindle cell carcinoma

## Abstract

Pleuropulmonary spindle cell carcinoma (SpCC) is a very rare tumor that belongs to a subgroup of sarcomatoid carcinomas of non‐small cell carcinomas of the lung. Breast carcinoma is one of the most common malignancies associated with metachronous second primary cancers. To our knowledge, this is the first report of a rare case of multiple pleuropulmonary SpCC in a 70‐year‐old woman, 16 years after mastectomy and chemotherapy for breast carcinoma.

**Summary**

- Pleuropulmonary spindle cell carcinoma (SpCC) can manifest as a metachronous primary tumor years after treatment for breast carcinoma, highlighting the necessity for long‐term vigilance in cancer survivors.

## 1 Introduction

Spindle cell carcinoma (SpCC) cases are rare tumors, accounting for only 0.2%–0.3% of all lung cancers [1]. Pleural SpCC is even rarer; in a review of 86 cases, pleural disease was not identified [2].

Metachronous primaries are cancers that develop 6 months after the diagnosis of the first primary malignancy. Herein, we report a 70‐year‐old woman with pleural SpCC who had a history of mastectomy for breast cancer 16 years earlier.

## 2 Case History/Examination

In July 2018, a 70‐year‐old woman was referred to our clinic with complaints of dyspnea and right pleuritic chest pain. She had never smoked. She had a history of left mastectomy due to adenocarcinoma in 2002. Additionally, she had undergone chemotherapy and left‐side radiotherapy for breast carcinoma. She had been well for 17 years without any symptoms or metastasis.

## 3 Methods

### 3.1 Differential Diagnosis

Given the patient's history and presentation with large pleural masses, several differential diagnoses were considered. Pulmonary metastases from the patient's prior breast carcinoma were a primary concern, as metastases often present with pleural involvement. *Echinococcus granulosus* infection was also considered given the patient's rural background and exposure to sheep farming; however, ultrasonography and CT scan findings were inconsistent with hydatid disease.

Tuberculosis was considered as well, given the pleural involvement and patient's symptoms. However, the absence of typical radiographic features such as cavitations and lack of granulomatous inflammation on biopsy, along with a negative microbiological workup, made TB unlikely. Malignant mesothelioma was another differential diagnosis, but immunohistochemical staining helped distinguish between mesothelioma and spindle cell carcinoma, ruling out mesothelioma.

### 3.2 Investigations

**FIGURE 1:**
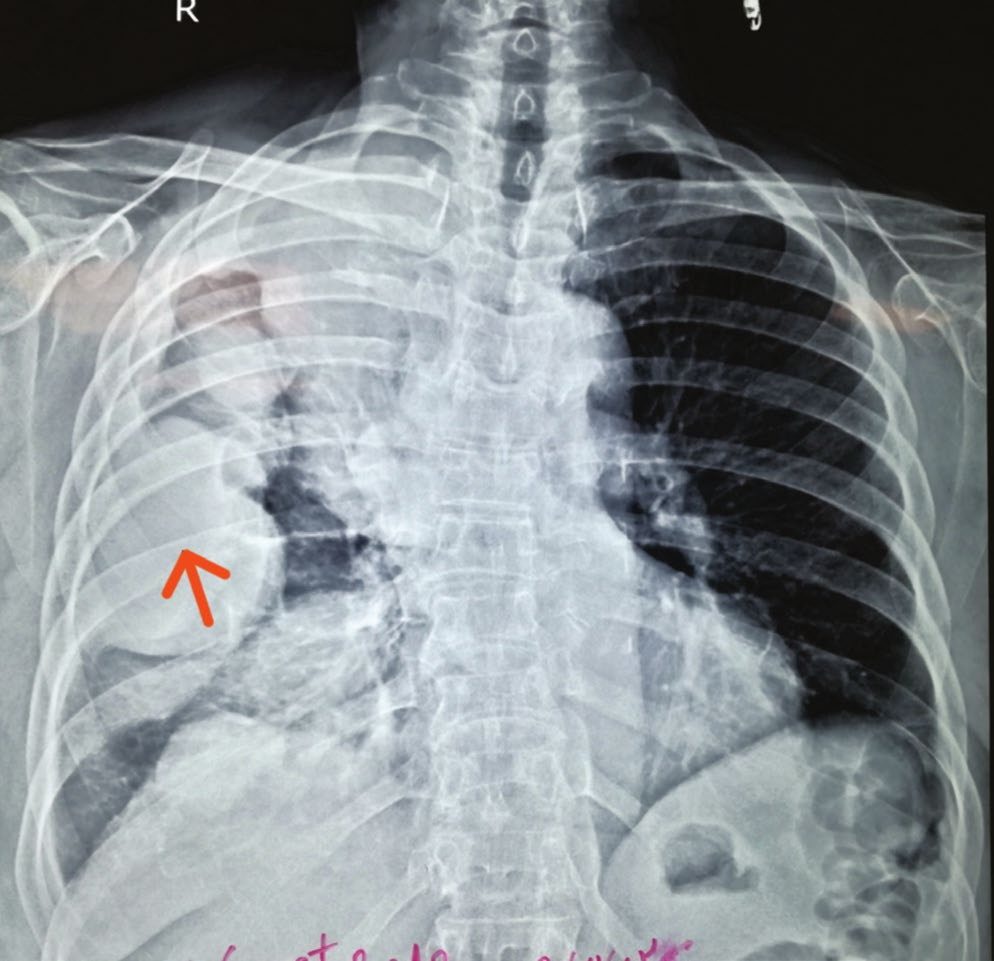
Chest X‐ray Findings. Clinical Question: Considering the patient's medical history, what is the likely diagnosis for the large, round masses observed on the right side of the chest X‐ray? Answer: The chest X‐ray reveals several large, round masses on the right side, which, given the patient's history of breast carcinoma and treatment 16 years earlier, are suggestive of pleuropulmonary spindle cell carcinoma (SpCC).

**FIGURE 2:**
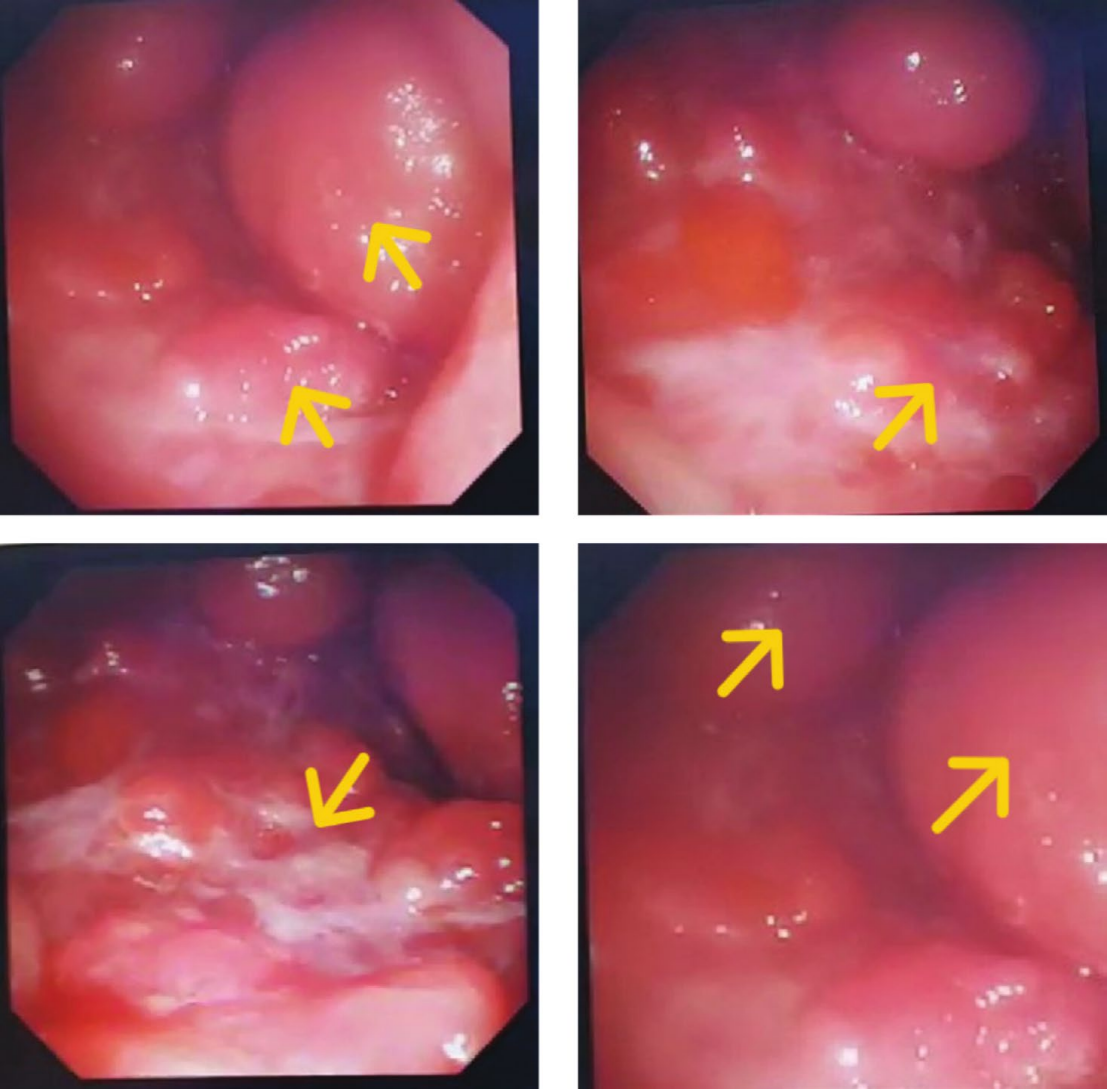
Thoracoscopy Findings. Clinical Question: What do the thoracoscopy images reveal about the nature of the masses observed in the patient's pleura? Answer: The semi‐rigid fiberoptic thoracoscopy with pleural biopsy revealed large solid masses with sharp borders. These findings are consistent with pleuropulmonary spindle cell carcinoma (SpCC), which, given the patient's medical history.

We immediately performed ultrasonography of the masses, which revealed solid masses incompatible with hydatosis. Considering the patient's medical history, we performed a chest x‐ray (Figure 1), which showed several large, round masses on the right side. The chest computerized tomography (CT) scan showed large solid masses. Additionally, semi‐rigid fiberoptic thoracoscopy with pleural biopsy revealed large solid masses with sharp borders (Figure 2). A moderate pleural effusion was noted on imaging. The analysis did not reveal malignant cells, and the effusion was likely reactive, secondary to the pleural masses. Light microscopic examination showed fragments of pleural tissue with areas of mesothelial cell hyperplasia and proliferation of spindle cells with nuclear atypia in loose fibrotic stroma, consistent with a spindle cell tumor. The results of immunohistochemical (IHC) analysis were positive for AE1/AE3, vimentin, partial WT1, Carlitin, and P53, but negative for estrogen receptor and mammaglobin.

## 4 Conclusions and Results

The final diagnosis was spindle cell sarcoma without a metastatic component from the breast. The patient's history of mastectomy and treatment for breast carcinoma 16 years earlier was taken into consideration, and the pleural spindle cell carcinoma (SpCC) was diagnosed through thorough differential diagnosis, imaging, biopsy, and immunohistochemical analysis. Follow‐up and outcomes were based on the diagnostic results, and the patient's condition was closely monitored to manage any further developments.

## 5 Discussion

We reported a rare case of metachronous spindle cell carcinoma (SpCC) 16 years after primary breast cancer. SpCC belongs to a group of neoplasms that are collectively called sarcomatoid carcinoma (SC). They exhibit properties of both epithelial and mesenchymal tumors. These rare malignancies account for only 0.52% of all the diagnosed 955,899 cases of non‐small‐cell lung carcinoma (NSCLC) [3]. The World Health Organization (WHO) [4] classified SC of lung into five major subtypes as follows: pleomorphic carcinoma, carcinosarcoma, SpCC, giant cell carcinoma, and pulmonary blastoma. Of the 95 patients studied by Ghrewati [5], there were six cases of spindle cell carcinoma, 29 cases of pleomorphic carcinoma, six cases of carcinosarcoma, and one case of giant cell carcinoma. Eighty were male, and 15 were female. The median patient age was 64 years (range: 43–80 years).

According to the definition by the WHO, “SpCC consists of an almost pure population of epithelial spindle cells, with no differentiated elements” [4, 6]. Most of these tumors can be classified by light microscopy alone. However, these tumors may be mistaken for carcinosarcomas or sarcomas. Immunohistochemistry (IHC) analysis contributes to an improved diagnosis of this spectrum of tumors [7].

In our patient, SpCC was diagnosed by light microscopy and confirmed by IHC. IHC in SpCC shows strong positive reactions for vimentin and cytokeratin and negative reactions for S100 protein, desmin, CD34, and α‐smooth muscle actin.

One of the differential diagnoses for these tumors is sarcomatoid malignant mesothelioma [8]. Clinical manifestations of sarcomatoid mesothelioma often include pleural effusions, pleural nodules, and pleural thickening. D2–40 and WT1 are positive in mesothelioma; however, in our case, D2–40 and WT1 were negative.

SpCC of the lung is more commonly seen as a peripheral lesion. There were large pleural‐based masses on the right side in our patient. Ghrewati reported a 73‐year‐old man with a massive pleural effusion secondary to SpCC [9].

Metachronous second primary cancers develop more than 6 months after the diagnosis of the first primary cancer [10]. Breast carcinoma is one of the most common cancers associated with second primary cancers [11]. Women being treated for breast cancer with tamoxifen have an increased risk of developing gynecological cancer. Alkylating agents increase the incidence of leukemia. The rate of lung cancer is high after radiation therapy due to breast carcinoma. However, to best our knowledge, the concurrence of breast cancer and SpCC has not been described in the literature so far. In this regard, it is essential to understand whether there is a relationship with previous breast carcinoma.

In addition to the lung, SpCC is rarely reported in the breast [12], oral cavity, larynx, uterus, kidney, prostate, and conjunctiva [13]. One possibility is that the patient had breast SpCC 16 years earlier. However, the pathology, course of the disease, and response to treatment are not compatible with breast SpCC. Is SpCC in this patient a late complication and carcinogenesis of chemotherapy and radiotherapy? The role of chemotherapy and radiotherapy in the pathogenesis of these tumors has not been reported so far.

It should also be noted that, in our patient, SpCC developed on the contralateral side to the mastectomy radiotherapy area, which makes the effect of radiotherapy in its pathogenesis unlikely.

## Author Contributions

**Mohammadhossein Rahimirad:** investigation, resources, supervision, writing – original draft. **Sara Daneshvar:** investigation, methodology, writing – review and editing. **Shaghayegh Rahimirad:** data curation, methodology, writing – original draft. **Monireh Halimi:** conceptualization, investigation, methodology. **Akbar Sharifi:** investigation, methodology, project administration, supervision.

## Ethics Statement

This study was conducted in accordance with the Declaration of Helsinki. The protocol was approved by the Ethics Committee of Tabriz University of Medical Sciences.

## Consent

Written informed consent has been obtained from the patient involved in this study.

## Conflicts of Interest

The authors declare no conflicts of interest.

## Data Availability

The data that support the findings of this study are available from the corresponding author, Akbar Sharifi, upon reasonable request.

## References

[1] M. Kontic , J. Stojsic , R. Stevic , V. Bunjevacki , B. Jekić , and V. Dobricic , “Could Spindle Cell Lung Carcinoma Be Considered and Treated as Sarcoma, According to Its Clinical Course, Morphology, Immunophenotype and Genetic Finding?,” Pathology Oncology Research 19, no. 1 (2013): 129–133. 22923000 10.1007/s12253-012-9562-4

[2] A. Weissferdt , N. Kalhor , A. M. Correa , and C. A. Moran , ““Sarcomatoid” Carcinomas of the Lung: A Clinicopathological Study of 86 Cases With a New Perspective on Tumor Classification,” Human Pathology 63 (2017): 14–26. 27993578 10.1016/j.humpath.2016.12.010

[3] M. Rahouma , M. Kamel , N. Narula , et al., “Pulmonary Sarcomatoid Carcinoma: An Analysis of a Rare Cancer From the Surveillance, Epidemiology, and End Results Database†,” European Journal of Cardio‐Thoracic Surgery 53, no. 4 (2017): 828–834. 10.1093/ejcts/ezx417 29240878

[4] E. Brambilla , W. D. Travis , T. V. Colby , B. Corrin , and Y. Shimosato , “The New World Health Organization Classification of Lung Tumours,” European Respiratory Journal 18, no. 6 (2001): 1059–1068. 11829087 10.1183/09031936.01.00275301

[5] N. A. Karim , J. Schuster , I. Eldessouki , et al., “Pulmonary Sarcomatoid Carcinoma: University of Cincinnati Experience,” Oncotarget 9, no. 3 (2018): 4102–4108. 29423107 10.18632/oncotarget.23468 PMC5790524

[6] W. D. Travis , E. Brambilla , A. P. Burke , A. Marx , and A. G. Nicholson , Pathology and Genetics of Tumours of the Lung, Pleura, Thymus and Heart (IARC Press, 2015). 10.1097/JTO.0000000000000663 26291007

[7] X. Li , D. Wu , H. Liu , and J. Chen , “Pulmonary Sarcomatoid Carcinoma: Progress, Treatment and Expectations,” Ther Adv Med Oncol 12 (2020): 1758835920950207. 32922522 10.1177/1758835920950207 PMC7450456

[8] K. Kushitani , Y. Takeshima , V. J. Amatya , O. Furonaka , A. Sakatani , and K. Inai , “Differential Diagnosis of Sarcomatoid Mesothelioma From True Sarcoma and Sarcomatoid Carcinoma Using Immunohistochemistry,” Pathology International 58, no. 2 (2008): 75–83. 18199156 10.1111/j.1440-1827.2007.02193.x

[9] M. Ghrewati , A. Mahmoud , M. Mohtadi , et al., “Spindle Cell Carcinoma Presenting as a Massive Pleural Effusion,” Cureus 16, no. 2 (2024): e54526. 38516459 10.7759/cureus.54526 PMC10956378

[10] K. Lei , X. He , L. Yu , et al., “Breast Cancer Prognosis Is Better in Patients Who Develop Subsequent Metachronous Thyroid Cancer,” PLoS One 14, no. 5 (2019): e0215948. 31042767 10.1371/journal.pone.0215948 PMC6493754

[11] N. Sharma , J. L. Thiek , D. Rituparna , J. Mishra , and A. S. Singh , “Metachronous Cancer of Breast and Adenocarcinoma of Cervix: A Rare Case Report,” J Menopausal Med 23, no. 2 (2017): 131–134. 28951862 10.6118/jmm.2017.23.2.131 PMC5606911

[12] E. A. Rakha , E. Brogi , I. Castellano , and C. Quinn , “Spindle Cell Lesions of the Breast: A Diagnostic Approach,” Virchows Archiv 480, no. 1 (2022): 127–145. 34322734 10.1007/s00428-021-03162-x PMC8983634

[13] Y. L. Chang , Y. C. Lee , J. Y. Shih , and C. T. Wu , “Pulmonary Pleomorphic (Spindle) Cell Carcinoma: Peculiar Clinicopathologic Manifestations Different From Ordinary Non‐Small Cell Carcinoma,” Lung Cancer 34, no. 1 (2001): 91–97. 11557118 10.1016/s0169-5002(01)00224-0

